# Integration analysis of miRNA-mRNA pairs between two contrasting genotypes reveals the molecular mechanism of jujube (*Ziziphus jujuba* Mill.) response to high-temperature stress

**DOI:** 10.1186/s12870-024-05304-0

**Published:** 2024-06-27

**Authors:** Juan Jin, Lei Yang, Dingyu Fan, Lili Li, Qing Hao

**Affiliations:** 1 Institute of Horticulture Crops, Xinjiang Academy of Agricultural Sciences, Urumqi, Xinjiang 830091 China; 2 The State Key Laboratory of Genetic Improvement and Germplasm Innovation of Crop Resistance in Arid Desert Regions (Preparation), Urumqi, Xinjiang 830091 China; 3 Key Laboratory of Genome Research and Genetic Improvement of Xinjiang Characteristic Fruits and Vegetables, Urumqi, Xinjiang 830091 China

**Keywords:** *Ziziphus jujuba* Mill, miRNAs, mRNAs, High-temperature stress, Regulatory network, Functional enrichment analysis

## Abstract

With global warming, high temperature (HT) has become one of the most common abiotic stresses resulting in significant crop yield losses, especially for jujube (*Ziziphus jujuba* Mill.), an important temperate economic crop cultivated worldwide. This study aims to explore the coping mechanism of jujube to HT stress at the transcriptional and post-transcriptional levels, including identifying differentially expressed miRNAs and mRNAs as well as elucidating the critical pathways involved. High-throughput sequencing analyses of miRNA and mRNA were performed on jujube leaves, which were collected from “Fucumi” (heat-tolerant) and “Junzao” (heat-sensitive) cultivars subjected to HT stress (42 °C) for 0, 1, 3, 5, and 7 days, respectively. The results showed that 45 known miRNAs, 482 novel miRNAs, and 13,884 differentially expressed mRNAs (DEMs) were identified. Among them, integrated analysis of miRNA target genes prediction and mRNA-seq obtained 1306 differentially expressed miRNAs-mRNAs pairs, including 484, 769, and 865 DEMIs-DEMs pairs discovered in “Fucuimi”, “Junzao” and two genotypes comparative groups, respectively. Furthermore, functional enrichment analysis of 1306 DEMs revealed that plant-pathogen interaction, starch and sucrose metabolism, spliceosome, and plant hormone signal transduction were crucial pathways in jujube leaves response to HT stress. The constructed miRNA-mRNA network, composed of 20 DEMIs and 33 DEMs, displayed significant differently expressions between these two genotypes. This study further proved the regulatory role of miRNAs in the response to HT stress in plants and will provide a theoretical foundation for the innovation and cultivation of heat-tolerant varieties.

**Supplementary Information:**

The online version contains supplementary material available at 10.1186/s12870-024-05304-0.

## Introduction

Environmental factors are crucial in regulating plant growth, development, and defense responses [1]. With global warming, HT stress possibly results in significant crop yield losses, a great concern in agriculture. HT stress induces cell membrane damage, affects cell division and protein synthesis, and disrupts cell homeostasis, which results in a negative influence on plants, including leaf chlorosis, severe retardation in growth and development, increased risk of disease, and even death [2, 3]. Thus, exploring the underlying molecular mechanisms of plant responses to HT stress is vital to ensure crop yield.

Advanced development of next-generation sequencing technology and bioinformatics have facilitated the molecular mechanism investigation of plant response to HT stress based on miRNA-mRNA analyses [4]. MicroRNAs (miRNAs) are short endogenous non-coding RNAs that serve as post-transcriptional regulators to modulate the expression levels of functional target genes (mRNA) [5], which play crucial roles in controlling major biological processes in plants [6]. Hao et al. indicated that the up-regulated miR172c-3p, miR395a, miR397a, miR408-5p, and miR827 might be involved in HT stress response in herbaceous peony at the post-transcriptional level [7]. After a comprehensive analysis of miRNAs-mRNAs of maize exposed to HT stress, Zhang et al. pointed out that miR164 is the regulator of maize to HT stress response at the post-transcriptional level [8]. It follows that integrated analysis of miRNA-mRNA can clarify the regulatory role of miRNAs under stress conditions and define the essential position of miRNA in regulating plant stress resistance, thus providing novel insights into exploring the mechanisms and methods of improving stress resistance in heat-sensitive plants [9].

As a deciduous fruit tree native to China with over 3000 years of cultivation history and distributed worldwide, jujube (*Ziziphus Jujuba* Mill.) has recently harvested more than four million tons of jujube fruits in China every year [10], accounting for over 90% of global production. Notably, due to the unique climate and soil conditions in Xinjiang, China, such as adequate illumination and significant temperature difference between day and night, contributing to the better quality of jujube fruit, jujube has been widely planted in Xinjiang (over 400 thousand hectares) [11]. The Jujube industry has been the core of the development of the agricultural economy in Xinjiang, the primary production area of the Chinese jujube industry. However, in recent years, summer air temperature in Xinjiang has hit historic records continuously, which has resulted in adverse effects on the quality of jujube, even the production of jujube fruits. For instance, ambient temperatures above 30 °C during the blooming stage of jujube negatively influence pollination, fertilization, and fruit setting. Therefore, investigating the heat resistance mechanisms in jujube is crucial for cultivating heat-tolerant jujube crops. “Fucuimi” and “Junzao” are two jujube cultivars mainly planted in Xinjiang. Our previous investigations indicate that “Fucuimi” displays stronger fruit-setting ability and more stable yield under extremely high-temperature conditions but produces smaller jujube fruits than “Junzao”. So, exploring the mechanisms of heat tolerance in jujube, aiming to cultivate heat-tolerant varieties, is of great significance for the agricultural sector.

To explore the underlying mechanism of miRNAs modulating the expression levels of functional mRNAs in different genotypes of jujube exposed to HT stress, an integrated analysis of miRNA-mRNA was performed on two different jujube cultivars (heat-tolerant genotype, “Fucuimi” and heat-sensitive genotype, “Junzao”) subjected to heat stress. The strategy of this study is (1) analysis of miRNA and mRNA profile to identify DEMIs and DEM, (2) identification of DEMI-target DEM pairs responsive to HT stress, and (3) functional annotation and enrichment analysis of DEMIs target gene. This study provided a theoretical foundation and industry guidance for innovating and cultivating heat-tolerant varieties.

## Materials and methods

### Plant growth conditions, heat treatment and RNA extraction

The experiments were carried out in the Experimental Greenhouse of the Institute of Horticultural Crops, Xinjiang Academy of Agricultural Sciences (Xinjiang, China) from June 23 to June 30, 2021. The two-year-old pot-planted seedlings of Ziziphus jujuba Mill. varieties “Fucuimi” (heat-tolerant genotype) and “Junzao” (heat-sensitive genotype, ZJ) with similar canopy sizes (plant height 80 cm; canopy width 50–70 cm; trunk circumference 6 cm) were grown in a controlled experimental greenhouse with 100 µmol m^− 2^ s^− 1^ of photosynthetically active radiation and a 16 h/8 h-light/dark cycle. The “Fucuimi” and “Junzao” were exposed to 42 °C/30°C (day/night) and around 30% humidity to conduct HT stress treatments for 0, 1, 3, 5, and 7 d, named F0, F1, F3, F5, F7, J0, J1, J3, J5, and J7, respectively. The environmental conditions in the summer of Turpan (Xinjiang, China) and jujube characteristics were considered when choosing the experimental heat stress conditions. During the experimental period, the trees were irrigated with water once a day (400 mL/tree) at 20:00 to prevent drought stress and were irrigated with 1/2 Hoagland solution (200 mL/tree) once in two days. Three biological replications of collected leaves were included at each time point. A total of 30 samples were immediately frozen in liquid nitrogen and stored at −80 °C until analysis.

RNA extraction was performed according to previous studies [12, 13]. In brief, according to the manufacturer’s instructions, total RNA was extracted from the samples using a TRIzol™ reagent kit (Thermo Fisher Scientific, Waltham, MA, USA). The leaf samples (80 mg) were homogenized with 1 mL TRIzol reagent. The NanoDrop 2000 (Thermo Fisher Scientific, Wilmington, DE, USA) was used to determine the RNA concentration and purity. The RNA Nano 6000 Assay Kit of the Agilent Bioanalyzer 2100 system (Agilent Technologies, Santa Clara, CA, USA) was used to assess the RNA integrity. The RNA samples with an RNA integrity number (RIN-value) ≥ 8 and 1.8 < OD_260_/OD_280_ ratio < 2.0 were used. The RNA with RIN values from 7 to 10 indicates availability, and the RIN value is positively correlated with RNA quality and integrity.

### Small RNA library construction and sequencing

Library preparation for sRNA sequencing was constructed from total RNA samples sent to BioMarker (Beijing, China). Briefly, 3′- and 5′-RNA adaptors were ligated to sRNAs and then followed by RT-PCR. Small RNAs, ranging in length from 18 to 30 nt, were enriched by polyacrylamide gel electrophoresis (PAGE), and the PCR products were purified by the AMPure XP system (Beckman Coulter, Beverly, MA, USA). High-throughput sequencing of sRNA (single-end, 50 bp) was performed using an Illumina Hiseq platform (San Diego, CA, USA) according to the manufacturer’s recommended protocol.

Raw data (raw reads) of FASTQ format were firstly processed using an in-house program to obtain clean reads by removing reads containing adapter, reads containing poly-N, low-quality reads, and reads shorter than 18 nt and longer than 30 nt. The downstream analyses were all based on clean reads with high quality.

### miRNA expression analysis

Raw sRNA data were filtered to get clean reads. The unannotated reads of miRNA were obtained by filtering ribosomal RNA (rRNA), transfer RNA (tRNA), small nuclear RNA (snRNA), small nucleolar (snoRNA) and repeats, which were then mapped to *Ziziphus_jujuba*.GCF_000826755.1 genome. The unannotated reads of miRNA and the mapped reads were all obtained using Bowtie (http://sourceforge.net/projects/bowtie-bio/files/). For the identification of known miRNAs, all mapped reads were aligned with the known mature miRNAs (Species studied) in miRbase 22.0 database (http://www.mirbase.org/), allowing at most one mismatch. The novel miRNAs were predicted using miRDeep2 software based on the biological characteristics of miRNA [14]. Based on the miRNA expression in each sample, the miRNA expression level was normalized according to transcripts per million (TPM) [15]. Differential expression analysis was performed using the DESeq2 package in the R statistical software. The *P*-values were adjusted using the methods of Benjamini and Hochberg to control the false discovery rate (FDR). The miRNAs with *P*-adjusted value ≤ 0.05 were considered as differentially expressed genes in the analyzing system.

### Prediction and annotation of differentially expressed miRNAs targets

The TargetFinder software was used to predict the potential target genes of differentially expressed miRNAs according to the methods previously reported [16]. The predicted potential target genes of differentially expressed miRNAs were aligned with GO database (http://www.geneontology.org/) and KEGG database (http://www.genome.jp/kegg/) using BLAST software to annotate potential target genes.

### mRNA library construction and sequencing

Library preparation for transcriptome sequencing was constructed using NEBNext Ultra™ RNA Library Pre Kit following the manufacturer’s recommendations. The cDNA synthesis was conducted according to the previous study [17]. Oligo (dT) was used to enrich mRNA, and then cDNA was reversely transcribed using random primers with short segments as templates to construct a library. The cDNA fragments with 240 bp in length were preferentially selected by purifying library fragments with the AMPure XP system, which were then enriched by PCR. Sequencing of mRNA was performed on the Illumina Hiseq 2500 platform at BioMarker. Raw data (raw reads) of FASTQ format were conducted with a series of standardized processing to get high-quality clean reads. The downstream analysis was performed on the specific length range selected from clean reads.

### mRNA expression analysis

The clean reads of mRNA were mapped to the reference genome [18] by HISAT2 software (http://ccb.jhu.edu/software/hisat2/index.shtml). The novel mRNAs and genes were explored and annotated by String Tie and DIAMOND software, respectively. The annotation of mRNA was based on GO and KEGG databases. The gene expression levels were estimated by fragments per kilobase per million mapped reads (FRKM). Differential expression analysis of two groups was conducted using DESeq2 software. Considering the large number of identified mRNAs, genes with |log_2_(FC)| ≥ 1 and the *P*-adjusted value < 0.01 were considered differentially expressed mRNAs in the analyzing system.

### Functional annotation of DEGs and enrichment analysis

The GO enrichment analysis, which was conducted with GOseq R packages and clusterProfiler software, contributed to identifying the main biological functions of the DEGs. Besides, KEGG pathways and enrichment analysis of differentially expressed genes were implemented by KOBAS [19], which were performed to explore the biological functions of the potential targets involved [20].

### Quantitative real-time PCR analysis

As described by Bu et al., qRT-PCR was performed on RNA extracts from leaf samples of both cultivars at the five heat treatment time points using the ZjActin as the internal control to normalize the expression data [21]. Primers used for qRT-PCR are listed in Table S1. The RNA samples used for transcriptome sequencing were also used for qRT-PCR verification. Three biological replicates were mixed into one sample in equal amounts, and three technical replicates were performed. According to the previous study with some modifications [22], fluorescence quantitative PCR was performed according to the SYBR^®^ Green PCR Master Mix kit (Applied Biosystems, GA, USA), and the reaction system volume was 20 µL. The PCR procedure was as follows: initial incubation at 95 °C for 15 min, followed by 40 cycles of denaturing at 95 °C for 10 s, annealing at 55 °C for 20 s, and extension at 72 °C for 30 s. The relative gene expression level was calculated by the 2^−ΔΔCt^ method.

### Statistical analysis

SPSS 20.0 statistical software (SPSS, IL, USA) was used for one-way analysis of variance (ANOVA). Duncan’s multiple test was used to analyze the differences between the mean values, and the results were considered significant at a *P*-value ≤ 0.05. Data plotting was performed using GraphPad Prism 8.

## Results

### Quality evaluation of miRNA and mRNA sequencing data

To explore and elucidate the underlying molecular mechanisms of the *Ziziphus jujuba* Mill. response to HT stress, two cultivars, specifically the temperature-tolerant genotype “Fucuimi” and the temperature-sensitive genotype “Junzao”, were selected. Both cultivars were subjected to high-temperature treatment for 0, 1, 3, 5, and 7 days, and subsequently, three biological replicates of leaves were collected. To comprehensively understand the gene expression patterns in response to HT stress, 30 small RNA libraries, and 30 mRNA libraries were constructed. The Illumina Hiseq platform was employed for high-throughput sequencing analysis.

### Summary of the small miRNA library dataset

A total of about 1.25 × 10^7^ to 2.35 × 10^7^ raw reads and 1.03 × 10^7^ to 1.79 × 10^7^ clean reads were obtained from miRNA sequencing, respectively (Table 1). The Q30 bases percentage of raw reads was greater than 96.83%. The clean reads from each sample were mapped to *Ziziphus_jujuba*.GCF_000826755.1 genome, for which the mapped ratio varied from 72.63 to 79.82%. The above results demonstrated the good quality of the sequences, which could be used for further analysis. As shown in TableS 1 and S2, the numbers of known and novel miRNAs were summarized, and a total of 527 miRNAs were identified, including 45 known miRNAs and 482 novel miRNAs.

**Table 1:** Summary of miRNA sequencing dataset

Type	Fucumi^a^					Junzao^a^				
	0 d (F0)	1 d (F1)	3 d (F3)	5 d (F5)	7 d (F7)	0 d (J0)	1 d (J1)	3 d (J3)	5 d (J5)	7 d (J7)
Raw reads (×10^7^)	1.54±0.03	1.55±0.05	1.34±0.03	1.28±0.05	1.39±0.21	1.58±0.36	1.25±0.07	1.39±0.19	2.35±0.66	1.52±0.45
Clean reads (×10^7^)	1.16±0.05	1.24±0.03	1.12±0.06	1.09±0.05	1.17±0.17	1.38±0.35	1.04±0.05	1.03±0.01	1.79±0.69	1.28±0.33
%> Q30 (%)^b^	97.86±0.12	97.98±0.12	97.63±0.08	97.58±0.06	97.75±0.05	97.12±0.11	97.27±0.12	96.83±0.12	97.43±0.11	96.97±0.65
Mapped reads (×10^6^)	6.38±0.39	7.05±0.36	4.51±2.19	5.85±0.31	6.36±1.05	7.66±2.04	5.65±0.54	5.77±0.27	9.42±3.47	6.57±1.38
Mapped ratio (%)	78.87±0.86	76.08±1.90	72.98±0.93	73.16±1.28	72.63±0.54	79.82±0.71	77.55±4.14	79.10±2.34	73.77±1.26	72.80±2.32
Known miRNA	38±1	32±4	34±10	40±1	40±1	38±4	32±5	23±4	38±3	36±5
Novel miRNA	478±0	478±1	469±11	471±0	474±0	474±2	475±0	472±6	473±5	464±2

^a^Three biological replicates were collected and sequenced for each treatment, and the results were expressed as mean±standard deviation

^b^Q30 represents the percentage of bases with a Phred Quality Score greater than 30

Besides, 302 miRNAs belonging to 79 previously identified miRNA families (Table S3). The most dominant miRNA family identified in this study was MIR5291, containing 55 miRNA members, followed by MIR5770, MIR171_1, and MIR860 families, with 26, 19 and 17 miRNA members, respectively. As for the remaining 75 miRNA families, 33 families contained 2–14 members, while 42 families contained only one miRNA. Notably, the miRNAs, including novel_miR_404 and novel_miR_84, were only discovered in “Fucuimi”, and novel_miR_413, ppe-miR156b, novel_miR_136, novel_miR_378, and novel_miR_121 were only identified in “Junzao” (Table S2). In addition, ppe-miR156b was the most abundantly expressed miRNA in “Fucuimi” and “Junzao”.

### Summary of mRNA library dataset

A total of 224.58 GB of clean data from 30 samples were obtained by mRNA sequencing, and the clean data of each sample was more than 5.74 GB. As shown in Table 2, Q30 bases rates were greater than 94.21%, and the ranges of clean reads and GC content were 2.05 × 10^7^–2.92 × 10^7^ and 44.18–44.80%, respectively. The mapped ratio, obtained by aligning clean reads with a specific reference genome, ranged from 88.17 to 89.86%. The downstream analysis was performed on uniquely mapped clean reads ranging from 3.11 × 10^7^ to 4.50 × 10^7^. Table S4 showed that a total of 13,884 differentially expressed mRNAs (DEMs) were identified from 30 samples in response to HT stress.

**Table 2:** Summary of mRNA sequencing dataset

Type	Fucumi					Junzao				
	0 d (F0)	1 d (F1)	3 d (F3)	5 d (F5)	7 d (F7)	0 d (J0)	1 d (J1)	3 d (J3)	5 d (J5)	7 d (J7)
Clean reads (×10^7^)	2.49±0.41	2.68±0.14	2.05±0.16	2.65±0.04	2.92±0.60	2.36±0.30	2.62±0.07	2.38±0.30	2.49±0.37	2.47±0.24
Clean bases (×10^9^)	7.42±1.21	7.98±0.43	6.11±0.49	7.89±0.13	8.70±1.80	7.05±0.90	7.81±0.22	7.11±0.90	7.42±1.11	7.37±0.74
GC content (%)^a^	44.56±0.05	44.57±0.05	44.53±0.12	44.62±0.10	44.71±0.03	44.80±0.06	44.41±0.06	44.18±0.05	44.25±0.02	44.32±0.03
%> Q30 (%)^b^	94.41±0.31	94.21±0.18	94.46±0.20	94.24±0.16	94.59±0.23	94.54±0.22	94.59±0.12	94.37±0.08	94.42±0.16	94.56±0.01
Mapped reads (×10^7^)	4.47±0.74	4.76±0.24	3.61±0.30	4.72±0.09	5.21±1.06	4.24±0.54	4.66±0.12	4.22±0.53	4.41±0.67	4.39±0.45
Mapped ratio (%)	89.86±0.23	88.83±0.35	88.17±0.47	89.23±0.34	89.16±0.20	89.68±0.18	88.94±0.31	88.59±0.10	88.47±0.22	88.75±0.27
Uniquely mapped (×10^7^)	3.85±0.64	4.05±0.20	3.11±0.26	4.08±0.09	4.50±0.92	3.69±0.48	3.96±0.12	3.56±0.44	3.75±0.57	3.73±0.37
Uniquely mapped ratio (%)	77.41±0.30	75.62±0.80	75.81±0.85	77.13±0.55	77.04±0.08	78.02±0.33	75.51±0.32	74.75±0.31	75.21±0.22	75.39±0.91

The data were expressed as mean±standard deviation (*n* = 3)

^a^GC content of the bases

^b^Q30 represents the percentage of bases with a Phred Quality Score greater than 30

### Comparative analysis of DEMIs and DEMs in “Fucuimi” and “Junzao”

**Fig. 1:**
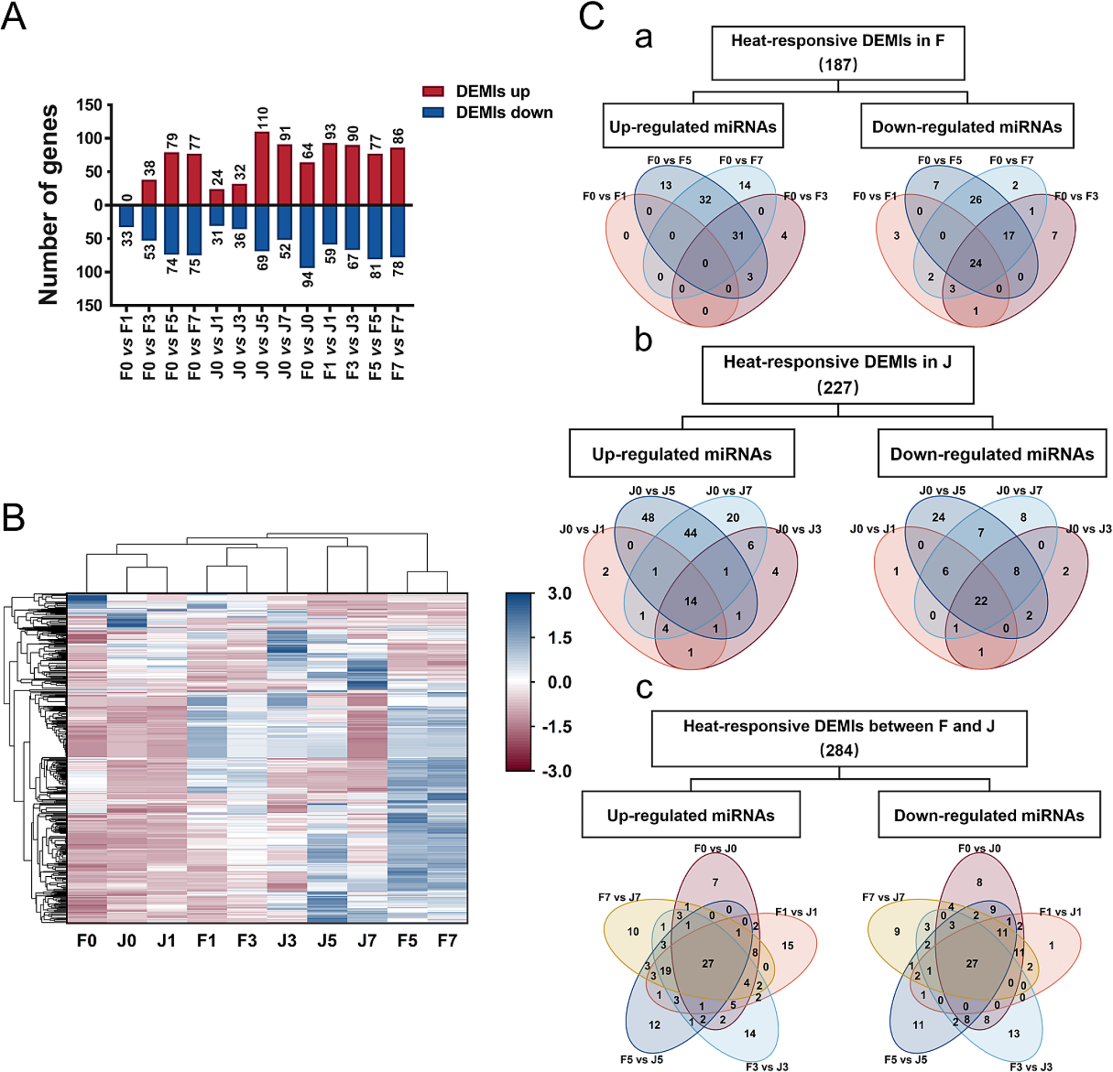
The expression profile of differentially expressed miRNAs (DEMIs) in *Ziziphus jujuba* leaves under high-temperature stress. (**A**) The number of DEMIs in two cultivars of jujube leaves under different treatment duration. (**B**) Heat map of all miRNAs expression profiles before and after high-temperature stress. (**C**) Venn diagrams of DEMIs at different treatment duration of the same cultivar or between two different cultivars at the same treatment time points. F0, F1, F3, F5, F7, J0, J1, J3, J5, and J7 represent the *Ziziphus jujuba* varieties “Fucuimi” and “Junzao” exposed to high-temperature treatment for 0, 1, 3, 5, and 7 days, respectively

**Fig. 2:**
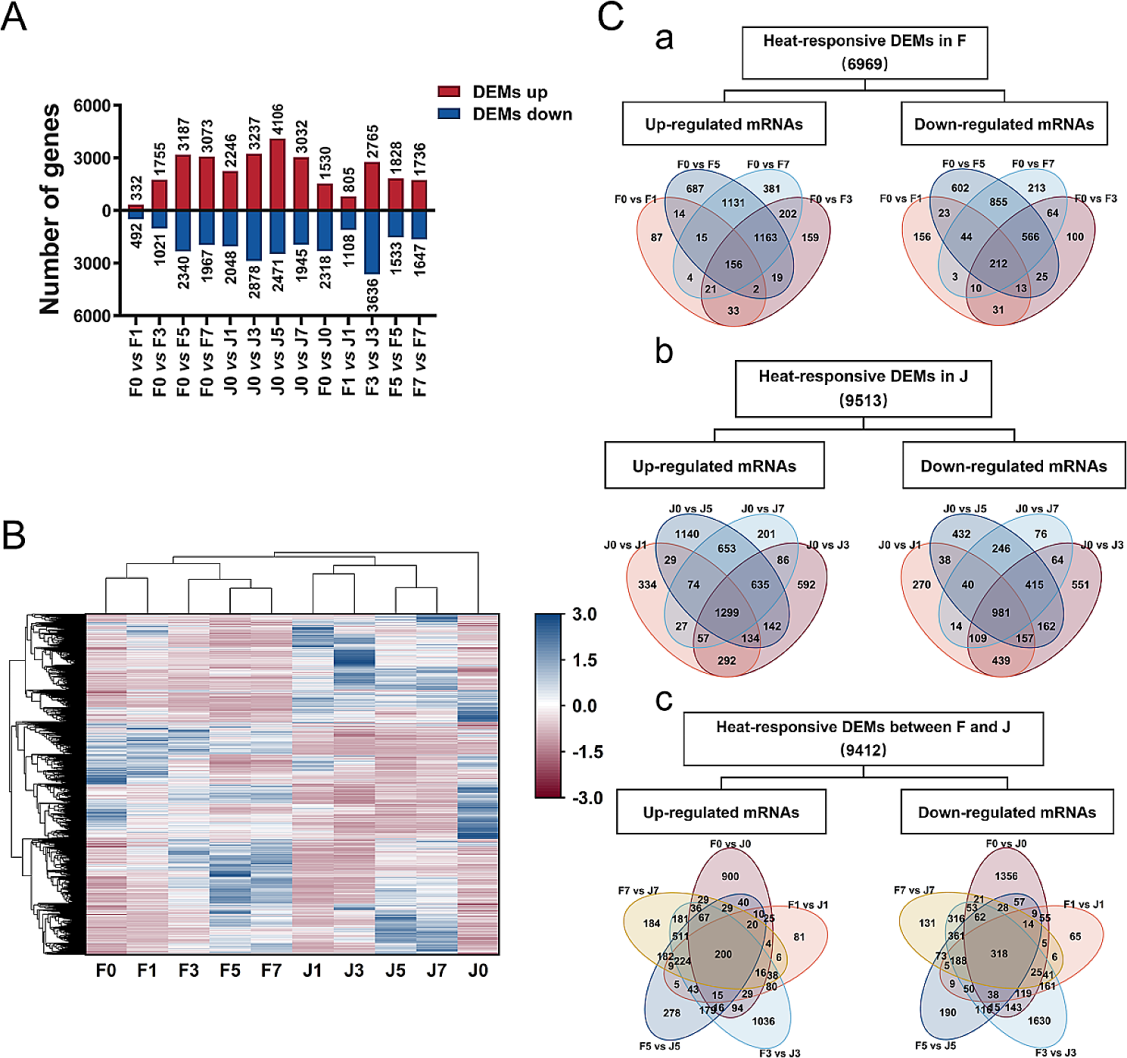
The expression profile of differentially expressed mRNAs (DEMs) in *Ziziphus jujuba* leaves under high-temperature stress. (**A**) The number of DEMs in two varieties of jujube leaves under different treatment duration. (**B**) Heat map of all DEMs expression profiles after high-temperature stress. (**C**) Venn diagrams of DEMs at different treatment duration of the same cultivar or between two different v cultivars at the same treatment time points. F0, F1, F3, F5, F7, J0, J1, J3, J5, and J7 represent the *Ziziphus jujuba* varieties “Fucuimi” and “Junzao” exposed to high-temperature treatment for 0, 1, 3, 5, and 7 days, respectively

The differences in DEMIs and DEMs expression patterns of two genotypes, “Fucuimi” and “Junzao” were analyzed to clarify the heat-tolerant capabilities of the two jujube cultivars at the gene level. As shown in Figs. 1 and 2, when “Fucuimi” and “Junzao” were exposed to high temperature, a total of 187 DEMIs and 6969 DEMs were identified in “Fucuimi” and a total of 227 DEMIs and 9513 DEMs were identified in “Junzao”. Besides, the DEMIs and DEMs expression levels of the two genotypes with the same treatment duration were compared. In the five comparison groups (F0 vs J0, F1 vs. J1, F3 vs. J3, F5 vs. J5 and F7 vs. J7), a total of 284 DEMIs and 9412 DEMs were determined, of which 54 miRNAs (27 up-regulated and 27 down-regulated) and 518 mRNAs (200 up-regulated and 318 down-regulated) were all differentially expressed in the five groups (Fig. 1C and 2C). Similar to “Fucuimi”, there was the least amount of DEMIs at 1 d (24 up-regulated and 31 down-regulated) and the largest amount of DEMIs at 5 d (110 up-regulated and 69 down-regulated) in “Junzao”. With the extension of HT stress duration, the number of DEMIs increased, and the number of DEMs in groups “Fucuimi” and “Junzao” first increased and then decreased. The cluster heatmap of all miRNA and DEM expression profiles illustrated that there were significant differences in miRNA and mRNA expression levels in samples before and after stress as well as in two genotypes with the same HT stress duration.

### Prediction and functional annotation of DEMIs-targeted genes

The TargetFinder software was used to predict and identify the potential target genes of DEMIs regulated by HT stress or differently expressed between two genotypes (Table S5). The results showed that 2139 target genes were predicted for 120 DEMIs from “Fucuimi”, 2470 target genes were predicted for 169 DEMIs from “Junzao”, and 2842 target genes were predicted for 163 DEMIs which were discovered differentially expressed between two genotypes (Table S4 and Fig. S1A). Besides, mRNA sequencing was applied to obtain the differentially expressed target genes (DEMs), of which 1306 DEMIs-DEMs pairs were obtained based on 141 DEMIs (Table S6), including 484, 769, and 865 DEMIs-DEMs pairs during HT stress from “Fucuimi”, “Junzao”, and between the two genotypes, respectively.

To explore the underlying mechanisms of DEMIs modulating the expression levels of functional DEMs and thus regulating HT stress, the GO and KEGG enrichment analyses were performed on 1306 DEMIs-targeted genes that were differentially expressed, aiming to clarify the potential functions of these DEMs and the pathways they are involved in. The GO annotation of these DEMs was classified into three categories (biological process, cellular component, and molecular function). The majority of the DEMs were involved in the metabolic process and cellular process of the biological process category, in the membrane and organelle terms of the cellular component category, and in the binding and catalytic activity of the molecular function category (Fig. S1B). In addition, KEGG enrichment analysis contributed to investigating the biochemical pathways that these DEMs were involved in. The KEGG enrichment analysis of 1306 DEMs was annotated into five categories (cellular processes, environmental information processing, genetic information processing, metabolism, and organismal systems). Compared with other pathways, more DEMs were enriched in plant-pathogen interaction, starch and sucrose metabolism, spliceosome, and plant hormone signal transduction pathways (Fig. S1C).

### Crucial pathway analysis of HT stress in *Ziziphus jujuba* Mill. leaves

There were 76, 40, 32, and 43 genes enriched in plant-pathogen interaction, starch and sucrose metabolism, spliceosome, and plant hormone signal transduction pathways, respectively, which were the top four enriched pathways of the DEMs and regulated the organismal system, metabolism, genetic information processing, and environmental information processing of the plants, respectively. To investigate the regulation mechanism of *Ziziphus jujuba* in response to HT stress, DEMIs and DEMIs target genes with |log_2_(FC)| ≥ 1 and *P* value ≤ 0.01 of the top four enriched pathways were chosen to analyze the differences in the miRNAs and mRNAs levels before and after stress as well as in the two genotypes “Fucuimi” and “Junzao” with the same HT stress duration (Table S7). Besides, the Person correlation analysis between DEMIs in all samples and their targeted gene expression levels of the top four KEGG enrichment pathways during high-temperature stress was also carried out (Fig. S2).

### Plant-pathogen interaction pathway

**Fig. 3:**
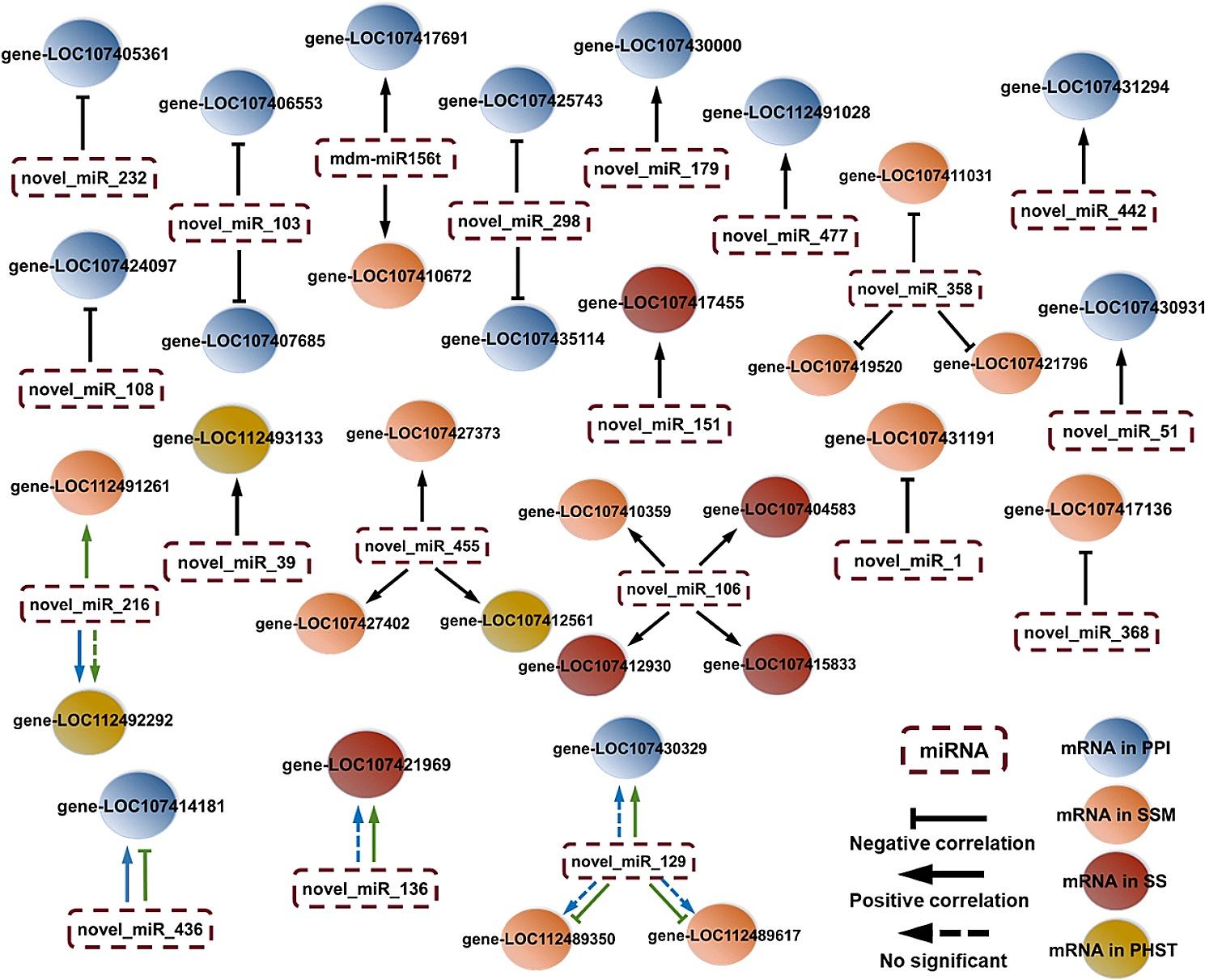
miRNA-mRNA correlation of *Ziziphus jujuba* network under high-temperature stress. The blue and green arrows represent the *Ziziphus jujuba* varieties “Fucuimi” and “Junzao”, respectively, and the black arrow represents the same regulatory trends of the two varieties. PPI, SSM, SS, and PHST represent the plant-pathogen interaction, starch and sucrose metabolism, spliceosome, and plant hormone signal transduction pathways, respectively

The most significantly differentially expressed 11 miRNAs and 13 mRNAs involved in the plant-pathogen interaction pathway were analyzed (Table S7 and Fig. S2). Five genes (LOC107430931, LOC107424097, LOC107425743, LOC107435114, and LOC112491028) related to disease resistance protein were up-regulated both in “Fucuimi” and “Junzao” after stress and were all differentially expressed between two genotypes (Table S7). The LOC107405361, related to the disease resistance protein RPS2, was down-regulated only in “Fucuimi” at 7 d after stress (Fig. 3). Three genes (LOC107414181, LOC107430000, and LOC107430329) related to WRKY transcription factor were all up-regulated in “Junzao” and differentially expressed in two genotypes. In contrast, LOC107414181 and LOC107430000 were down-regulated in “Fucuimi”. LOC107417691, related to the EIX receptor, was differentially expressed between two genotypes, and LOC107431294, related to cyclic nucleotide-gated channel, was up-regulated. As Fig. S2 presented, the genes related (LOC107406553 and LOC107407685) to serine/threonine-protein kinase Pto were all negatively associated with novel_miR_358 and novel_miR_368 (*P* < 0.05), which were all involved in starch and sucrose metabolism pathway, and positively correlated with mdm-miR156t and novel_miR_136 (*P* < 0.05).

### Starch and sucrose metabolism pathway

As shown in Table S7 and Fig. S2, the LOC107419520, LOC107427373, LOC107427402, LOC107431191, LOC112489350, LOC112489617, and LOC112491261 were all related to sucrose synthase, of which the LOC107427373, LOC107427402 and LOC107431191 were up-regulated after HT stress, and LOC112489350 and LOC112491261 were down-regulated and up-regulated in “Junzao”, respectively. Besides, LOC112491261 was not identified in “Fucuimi”, and these seven genes were all differentially expressed between two genotypes. The β-glucosidase (LOC107410359 and LOC107410672) and endoglucanase (LOC107417136) related genes were up-regulated, while the α-amylase (LOC107411031) and glucan endo-1,3-beta-glucosidase (LOC107421796) associated genes were down-regulated after stress. Fig. S2 showed that the novel_miR_358 was positively associated with LOC107411031 (α-amylase), LOC107419520 (sucrose synthase) and LOC107421796 (glucan endo-1,3-beta-glucosidase) (*P* < 0.05), and were negatively correlated with LOC107417136 (endoglucanase), LOC107427373 (sucrose synthase) and LOC107427402 (sucrose synthase) (*P* < 0.01).

### Spliceosome pathway

The heat shock protein-related genes (LOC107404583 and LOC107415833) were differentially expressed between two genotypes “Fucuim” and “Junzao” after HT stress. The ATP-dependent RNA helicase DHX8/PRP22 related genes (LOC107412930, LOC107417455, and LOC107421969) were significant differences in jujube leaves before and after stress. LOC107412930 was up-regulated both in “Fucuimi” and “Junzao” opposite to LOC107417455, and LOC107421969 was up-regulated only in “Junzao”.

### Plant hormone signal transduction pathway

In the plant hormone signal transduction pathway, the expression of protein TIFY 9 gene (LOC107412561) was up-regulated after 5 d stress in “Funcuimi” and after 1 d stress in “Junzao”. Also, the gene (LOC112493133) related to cytokinin receptor was up-regulated both in two genotypes after stress, while the gene (LOC112492292) related to systemin receptor SR160 (protein brassinosteroid insensitive 1) was down-regulated in “Fucuimi” after 7 d stress.

### Construction of miRNA-mRNA correlation network

To further investigate the key candidates for HT tolerance, a miRNA-mRNA correlation network was constructed to clarify the regulatory relationship between DEMIs and DEMs involved in the top four pathways in jujube leaves response to HT stress. Figure 3 showed that 33 DEMI target genes (from DEMs) were related to 20 DEMIs. The miRNAs, novel_miR_232, novel_miR_108, novel_miR_103, novel_miR_298, novel_miR_358, novel_miR_1, and novel_miR_368, were all negatively correlated with their target genes in “Fucuimi” and “Junzao” after stress, while mdm-miR156t, novel_miR_179, novel_miR_447, novel_miR_442, novel_miR_51, novel_miR_151, novel_miR_39, novel_miR_455, and novel_miR_106 were all positively correlated with their target genes in “Fucuimi” and “Junzao” after stress. However, the correlation between novel_miR_216, novel_miR_436, novel_miR_129, and novel_miR_136 and their target genes varied between the two genotypes after HT stress.

### qRT-PCR verification

To confirm the validity of the RNA-seq data, 6 DEGs were selected for real-time PCR analysis. The 6 DEGs were LOC107404976, LOC107432449, LOC107413540, LOC107415833, LOC107423628, LOC107414457, respectively. As shown in Fig. S3, the expression patterns of qRT‒PCR and RNA-seq data were consistent.

## Discussion

High temperature is one of the most common abiotic stresses that adversely affect the growth and development of crop plants like jujube grown in Xinjiang, China, resulting in poor morphology, physiology, and yield. Nevertheless, plants possess miRNA-regulated gene expression mechanisms that aid them in coping with high temperatures by regulating the expression of stress-related genes [23]. During HT stress, variations in miRNA levels in plants modify the expression level of their target mRNAs, which in turn influences protein accumulation, ultimately regulating the stress responses. In this study, detailed descriptions of the expression of miRNAs and mRNAs in jujube leaves of two genotypes (“Fucuimi” and “Junzao”) during HT stress were conducted.

Recently, the essential functions of miRNAs in exploring the regulation molecular mechanism of plant response to abiotic stress have garnered extensive attention [24]. As the post-transcriptional regulators that modulate the expression levels of functional target genes (mRNA) [5], miRNAs play crucial roles in controlling major biological processes in plants, including growth, development, and fitness to environment alteration [6]. It has been demonstrated that various miRNAs in plants are reported to be up or downregulated to cope with HT stress. Stief et al. reported that miR156 was highly induced after HT stress, which promoted sustained expression of HT stress-responsive genes [25]. After a comprehensive analysis of miRNA profiles of HT-sensitive and HT-tolerant cotton lines subjected to HT stress, Chen et al. suggested that miR160, miR167, and miR2949 families are the primary families that responded to HT stress at the sporogenous cell proliferation stage and miR3476 and miR393 were the dominant HT stress-responsive families at the pollen maturity stage [26]. Therefore, sRNA sequencing analysis was conducted in this study to investigate the underlying molecular mechanisms of miRNA regulation in the response of the jujube leaf system to HT stress. Among the 79 miRNA families identified, the MIR156, MIR164, MIR159, MIR166, MIR398, MIR396, MIR172, MIR319, and MIR168 families have been demonstrated to participate in HT stress responses of other plants in previous studies [3, 27–29]. The MIR319 family was reported to be associated with heat, cold, drought, salt stress, metal, and oxidative stresses in plants and is involved in regulating TCP family transcription factor (a group of genes encoding plant-specific transcription factors) related genes [3]. Apart from these proven miRNA families above, MIR2950, MIR482, MIR482, MIR828, MIR408, MIR171_1, and MIR815 families were predicted to play roles in jujube leaves response to HT stress (Table S7) and it is of great interest to explore the biological functions of them in plant HT stress.

Besides, transcriptome sequencing has been generally utilized to investigate the molecular mechanisms of plants adapting to stressful environments, such as HT stress, which can aid in identifying targets for molecular breeding to enhance crop stress tolerance [30]. For example, Eshel et al. utilized transcriptional profiling to reveal the heat adaptive features of the Brassicaceae desert model, *Anastatica hierochuntica* [31]. Hwang et al. comprehensively characterized Korean fir under heat stress using transcriptome analysis and identified 6401 DEMs, providing a crucial foundation for future studies on identifying HT-stress tolerant lines [32]. Since miRNAs regulate mRNA expression in plants through transcriptional repression [33], transcriptome sequencing was used in this study to detect the expression levels of miRNA target genes further to elucidate the regulatory functions of DEMIs under HT stress, thus providing a comprehensive view of the response regulators in jujube leaves during HT stress.

The functional annotations of DEMIs-targeted DEMs, based on GO and KEGG enrichment analysis, indicated that various metabolism pathways, organismal systems pathways, cellular processes, membrane components, and signal transduction responses participated in jujube leaf’s HT stress response. According to the KEGG enrichment analysis performed on the 1306 target DEMs of miRNAs in jujube leaves, this study focused on the top four enriched pathways, including plant-pathogen interaction, starch and sucrose metabolism, spliceosome, and plant hormone signal transduction pathways (Table S6 and Fig. S1). This result is consistent with the previous studies; for instance, Zhang et al. suggested that the mRNA profile of maize responses to HT stress is enriched in starch and sucrose metabolism and plant hormone signal transduction pathways [8]. The Gene Ontology classification analysis showed that high temperatures induce cell membrane damage and affect plant cell division, consistent with the result obtained by Go enrichment analysis in this study (Fig. S1B) and the results obtained by Ahmed et al. [28].

**Fig. 4:**
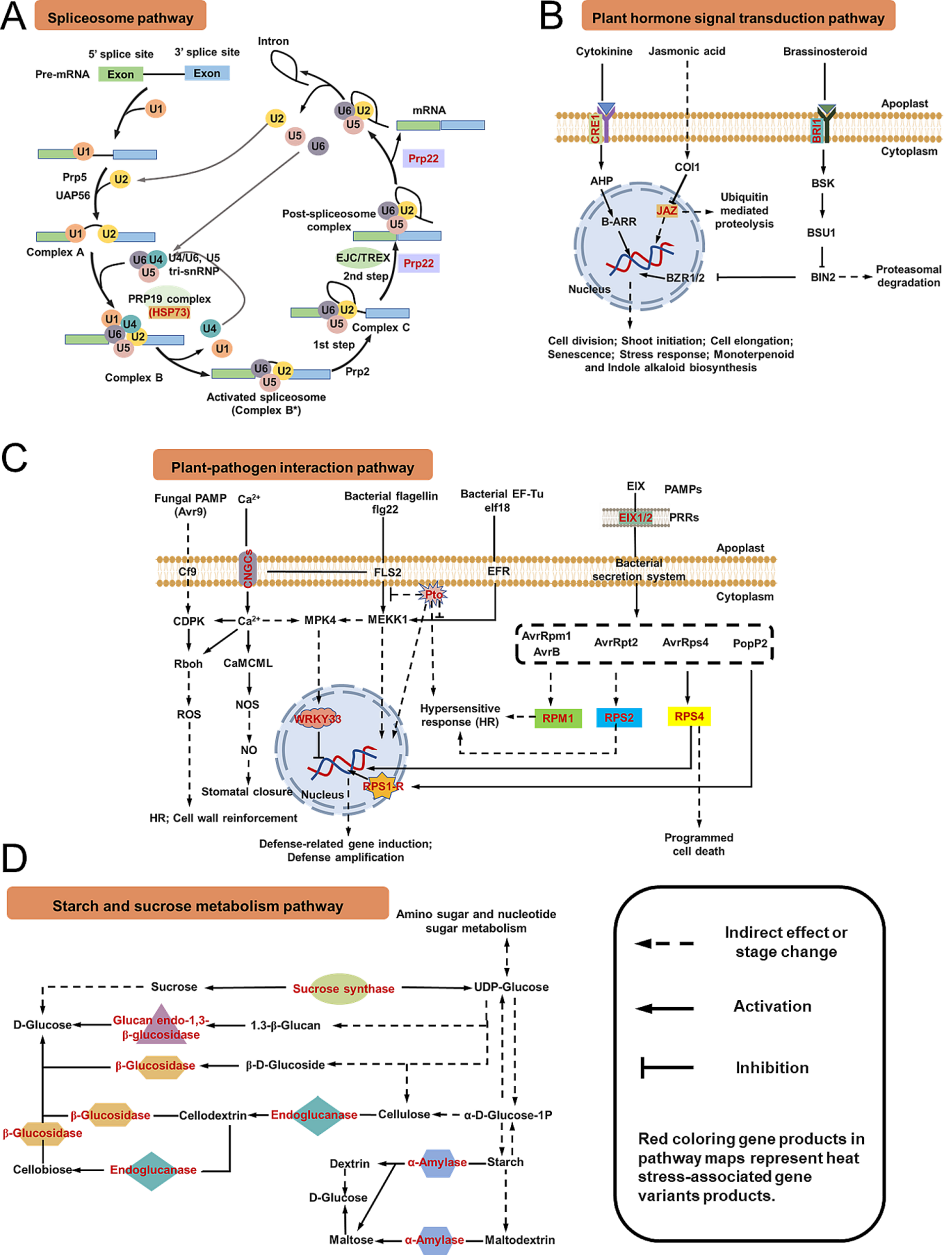
Crucial pathways of high-temperature stress response in jujube leaves of the “Fucuimi” and “Junzao” genotypes

Previous studies have demonstrated that HT stress can significantly impact plant-pathogen interactions and potentially increase the prevalence of insect vectors that transmit pathogens, resulting in an improvement in abiotic stress tolerance in plants [34]. In the present study, plant-pathogen interaction was the most enriched pathway by KEGG enrichment analysis in jujube leaves when responding to HT stress, which involved disease resistance protein RPS2, serine/threonine-protein kinase Pto, WRKY transcription factor 33/55, EIX receptor 1/2, disease resistance protein RPS4, disease resistance protein RPM1, cyclic nucleotide-gated ion channel, and disease resistance protein RPM1 related genes. Virus infection can induce significant physiological changes in host plants, such as reduced stomatal opening and lower transpiration rates, which lead to an elevation in leaf temperatures to minimize water loss [35]. Some studies have reported that specific viral and fungal endophytes can confer heat tolerance to their host plants [36, 37]. Calcium signal is a critical regulatory mechanism that enables crops to withstand abiotic stress [38]. The expression of the gene (LOC107431294, positively regulated by novel_miR_442) related to cyclic nucleotide-gated Ca^2+^ channel was significantly increased in “Junzao” after 7 d of HT stress, which indicated that the sensitivity of “Junzao” to calcium was enhanced in response to HT stress and potentially resulting in stomatal closure and a hypersensitive response (Fig. 4C). In plants, pathogen-associated molecular patterns (PAMPs) act as ligands for extracellular receptor proteins that activate signaling pathways leading to immunity [39], which are mediated by cell surface pattern recognition receptors (PRRs) [40]. The gene related to EIX 2 (PRRs) was differentially expressed between the two genotypes (Table S7). Plant resistance proteins (RPM1, RPS2, and RPS4) mediate resistance by recognizing the pathogen’s secreted effector proteins and triggering rapid responses [41]. The expression levels of RPM1 and RPS2 significantly differed between the two genotypes under HT stress. Pto is a serine/threonine protein kinase that plays a critical role in the hypersensitive cell death and activation of defense genes. The interaction between the product of the pathogen’s avirulence Pto gene and serine/threonine protein kinase is highly specific and sensitive to single amino acid changes in the Pto kinase, which may impede further advances by the pathogen [42]. The expression levels of Pto-related genes (both negatively regulated by novel_miR_103) were up-regulated in all HT-stressed samples of “Fucuimi” but almost down-regulated in “Junzao”, indicating that “Fucuimi” has stronger tolerance to HT stress than “Junzao”. The WRKY gene family has been demonstrated to regulate transcriptional reprogramming associated with plant stress responses by modulating plant secondary metabolites such as alkaloids and terpenes [43, 44]. WRKY33 is involved in regulating resistance to HT stress, but its expression is inhibited during high temperatures, increasing sensitivity to HT stress in plants [45]. The expression of the gene related to WRKY33 was up-regulated in “Junzao” but down-regulated in “Fucuimi” (Table S7), which was modulated by MIR172 family member novel_miR_436. In summary, the regulation of the plant-pathogen interaction pathway is critical for responding to HT stress in plants.

Starch and sucrose metabolism are physiological mechanisms contributing to plant resistance [46], which varies depending on the tissue, development stage, and external factors. Starch pools synthesized in photosynthetic source tissues and non-storge sinks have significantly enhanced overall plant productivity [47]. The disruptions in sucrose metabolism and carbohydrate concentrations resulting from HT stress can substantially augment crop yield losses [48]. The genes related to sucrose synthase, glucan endo-1,3-β-glucosidase, β-glucosidase, endoglucanase, and α-amylase involved in starch and sucrose metabolism pathway were differentially expressed in jujube leaves under HT stress (Table S7). Sucrose synthase is a glycosyl transferase enzyme that participates in sugar metabolism, especially in sink tissues [49]. The expression of sucrose synthase was regulated by seven DEMs modulated by novel_miR_358, novel_miR_455, novel_miR_1, novel_miR_129, and novel_miR_216. Differential regulation of sucrose synthase expression can significantly impact sucrose and UDP-glucose interconversion (Fig. 4D), and excessive accumulation of endogenous UDP-glucose may trigger programmed cell death [50]. All identified DEMs related to sucrose synthase were differentially expressed between the two genotypes. β-glucosidase, positively regulated by novel_miR_106 and mdm-miR156t in this study, catalyzes the hydrolysis of β-stereospecific bonds in oligosaccharides and glucosides, thus being involved in stress-related responses of plants [51]. Endoglucanase, a member of glycoside hydrolase family 5, plays a crucial role in the degradation of diverse plant polysaccharides [52], regulating the hydrolysis from cellulose to cellodextrin and from cellodextrin to cellobiose (Fig. 4D). The expression of the mRNA related to endoglucanase was negatively modulated by novel_miR_368 (Fig. 3).

Alternative splicing of precursor mRNA is a post-transcriptional regulatory process that modulates gene expression, thereby enhancing proteome diversity. This mechanism is often influenced by distinct plant developmental stages, environments, and genotypes [53]. In this work, the genes related to ATP-dependent RNA helicase Prp22 and heat shock proteins significantly differed in jujube leaves before and after HT stress and were also differentially expressed between two genotypes subjected to HT stress. The RNA helicase Prp22 plays an essential role in the release of the mRNA from the spliceosome during pre-mRNA splicing [54]. The expressions of Prp22-related genes in this study were regulated by novel_miR_106, novel_miR_151, and novel_miR_136 [55]. Besides, the alteration of protein structure caused by heat stress often negatively impacts protein function. The high expression levels of plant heat shock proteins (HSPs) are notably prominent in the process of heat stress acclimation, although they comprise only a tiny fraction of the thermal transcriptome in plants [56]. To mitigate damage to cellular proteins, the expression levels of HSPs are generally up-regulated and have been correlated with the acquisition of high-temperature tolerance in numerous instances, acting as chaperone proteins [57]. HSPs are broadly classified as HSP100, HSP70/40, HSP60/10 and sHSPs classes [57]. The gene (LOC107415833) related to HSP70, modulated by novel_miR_106, was up-regulated in “Fucuimi” after HT stress. Apart from this, in maize, heat shock determines the activation of alternate splicing mechanisms with a modified spliceosome able to operate at higher temperatures. Combined with previous studies, the results obtained in this study further suggested that HT stress conditions might have induced alternative splicing to increase the heat tolerance of plants.

Phytohormones are essential for various aspects of plant development, growth, and defense processes, particularly regulating the response to HT stress by preventing heat-induced damage and thus enhancing plant tolerance [58]. Clarifying the underlying molecular mechanisms associated with the regulation of heat response *via* plant hormone signal transduction pathways may provide a theoretical foundation for improving the heat tolerance of agriculturally significant crops. The genes related to CRE1, BRI1, and JAZ proteins, involved in plant hormone signal transduction pathways, were identified in jujube leaves responses to HT stress and were differentially expressed (Fig. 4). Jasmonate ZIM domain (JAZ) proteins, which are members of the TIFY gene family, play a critical role in the jasmonate (JA) signaling pathway in plants in response to abiotic stress. JAZ proteins, serving as core regulators and repressors of JA signaling, inhibit the expression of JA-responsive genes by suppressing the transcriptional activity of MYC2, MYC3, MYC4, and MYC5 [59]. The expression of the gene (LOC107412561) related to JAZ was up-regulated both in “Fucuimi” and “Junzao”, which was positively regulated by novel_miR_455 belonging to MIR171_1 family. The hybrid histidine kinase (CRE1) functions as a receptor for cytokinin, which is a class of phytohormones involved in various physiological processes and plant adaptations to temperature changes. Sobol et al. demonstrated that flower primordia of passion fruit (*Passiflora edulis*) genotypes with higher levels of cytokinin in their leaves can successfully reach anthesis during the summer season, suggesting a protective effect of cytokinin on developing flowers exposed to heat stress [60]. In response to HT stress, the CRE1 gene was up-regulated in jujube leaves of two genotypes and was found to be positively regulated by novel_miR_39. Brassinosteroids (BRs) are plant-derived steroid hormones activating a receptor complex at the cellular surface, comprising Brassinosteroid Insensitive1 (BRI1) and BRI1-associated kinase (BAK1), which positively regulate heat stress response [61]. In “Fucuimi,” the expression of BRI1 was found to be down-regulated, regulated by the target gene of novel_miR_216.

Furthermore, a regulatory DEMI-DEM network responding to HT stress was also constructed to investigate their potential interaction mechanisms based on DEMI and DEMI target genes with |log_2_(FC)| ≥ 1 and *P* value ≤ 0.01 involved in the top four enriched pathways. This could improve the understanding of the differences in heat-sensitive genotype and heat-tolerant genotype plants’ response to HT stress and also provide potential genes that could be utilized to enhance the heat-tolerant capacity in jujube plants. A comprehensive analysis of miRNA-mRNA regulatory networks under stress conditions can provide novel insights into exploring the mechanisms and methods of improving stress resistance in heat-sensitive plants [9]. Analyzing the interaction between miRNAs and mRNAs will contribute to understanding the regulatory role of miRNAs in plants’ responses to HT stress. Considering these DEMI-DEM pairs that are involved in the plant-pathogen interaction, starch and sucrose metabolism, spliceosome, and plant hormone signal transduction pathways played essential roles in the response of jujube subjected to HT stress, further research exploring and verifying the effects of HT stress on the specific miRNA and their target genes in jujube using molecular biology methods is necessary to gain more precise and comprehensive insights.

## Conclusion

In this study, small RNA and mRNA sequencing analyses were performed on jujube (*Ziziphus jujuba* Mill.) leaves collected from heat-tolerant (“Fucumi”) and heat-sensitive (“Junzao”) cultivars subjected to HT stress. The results illustrated that there were significant differences in miRNA and mRNA expression levels in samples before and after stress as well as in two genotypes. A comprehensive analysis of heat-responsive genes and miRNAs was carried out and revealed 1306 DEMIs-DEMs pairs under HT stress. Based on annotation and enrichment analysis, the 1306 DEMs were significantly enriched in plant-pathogen interaction, starch and sucrose metabolism, spliceosome, and plant hormone signal transduction pathways. In addition, integrated analysis of miRNA-mRNA revealed the regulatory roles of miRNA in the response to HT stress in jujube, thus providing novel insights into exploring the underlying molecular mechanisms of enhancing stress resistance of heat-sensitive plants.

### Electronic supplementary material

Below is the link to the electronic supplementary material.

Supplementary Material 1

Supplementary Material 2

Supplementary Material 3

Supplementary Material 4

Supplementary Material 5

Supplementary Material 6

Supplementary Material 7

Supplementary Material 8

## Data Availability

The raw sequence data reported in this paper have been deposited in the Genome Sequence Archive (Genomics, Proteomics & Bioinformatics 2021) in National Genomics Data Center (Nucleic Acids Res 2022), China National Center for Bioinformation/Beijing Institute of Genomics, Chinese Academy of Sciences (BioProject ID: PRJCA018436 and PRJCA016517) that are publicly accessible at https://ngdc.cncb.ac.cn/.
