# Supplementary material for: Integration analysis of miRNA-mRNA pairs between two contrasting genotypes reveals the molecular mechanism of jujube (Ziziphus jujuba Mill.) response to high-temperature stress
Source: BMC Plant Biol. 2024 Jun 27;24:612. doi: 10.1186/s12870-024-05304-0 (PMC11209981; doi:10.1186/s12870-024-05304-0)
Supplement: Supplementary file 1 — Supplementary Material 1 [file 12870_2024_5304_MOESM1_ESM.docx]

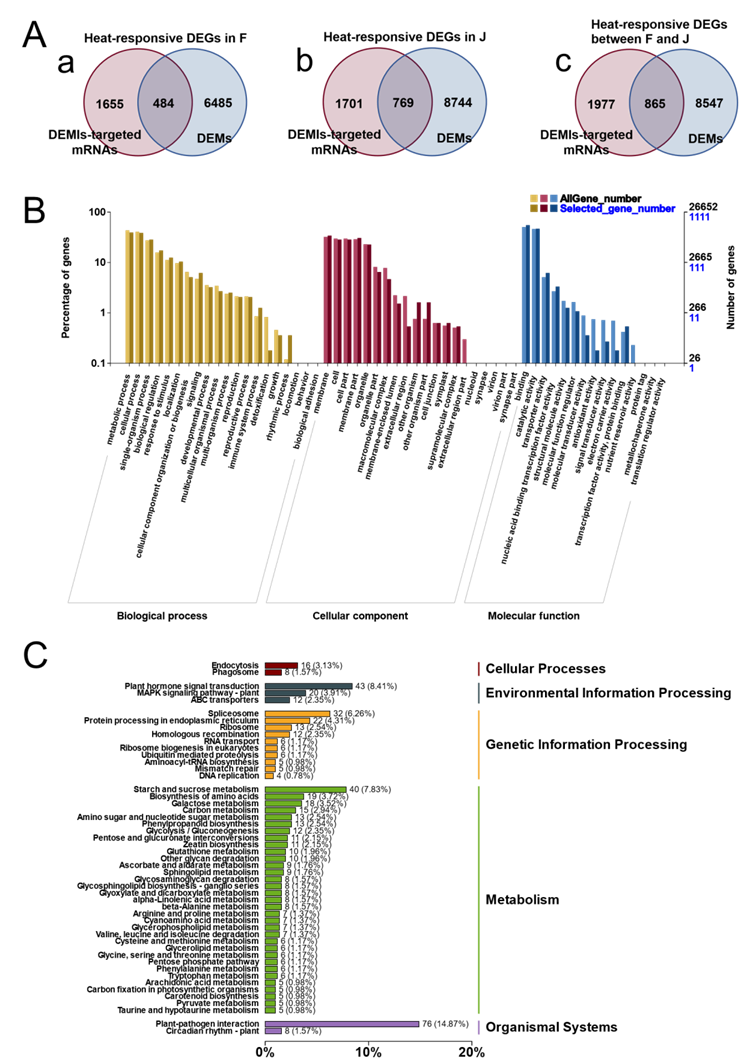


**Fig.S1** Analysis of differentially expressed miRNAs (DEMIs)-targeted genes and mRNAs (DEMs) during high-temperature stress of *Ziziphus jujuba* leaves. (A) Venn diagrams representing the numbers of DEMI-targeted genes and DEMs, and the number of DEMIs-DEMs pairs between two groups. (B) Gene Ontology classification analysis of identified differentially expressed DEMI-targeted genes (DEMs). (C) KEGG pathway enrichment for identified differentially expressed DEMI-targeted genes (DEMs). F and J represent the *Ziziphus jujuba* varieties “Fucuimi” and “Junzao”, respectively.


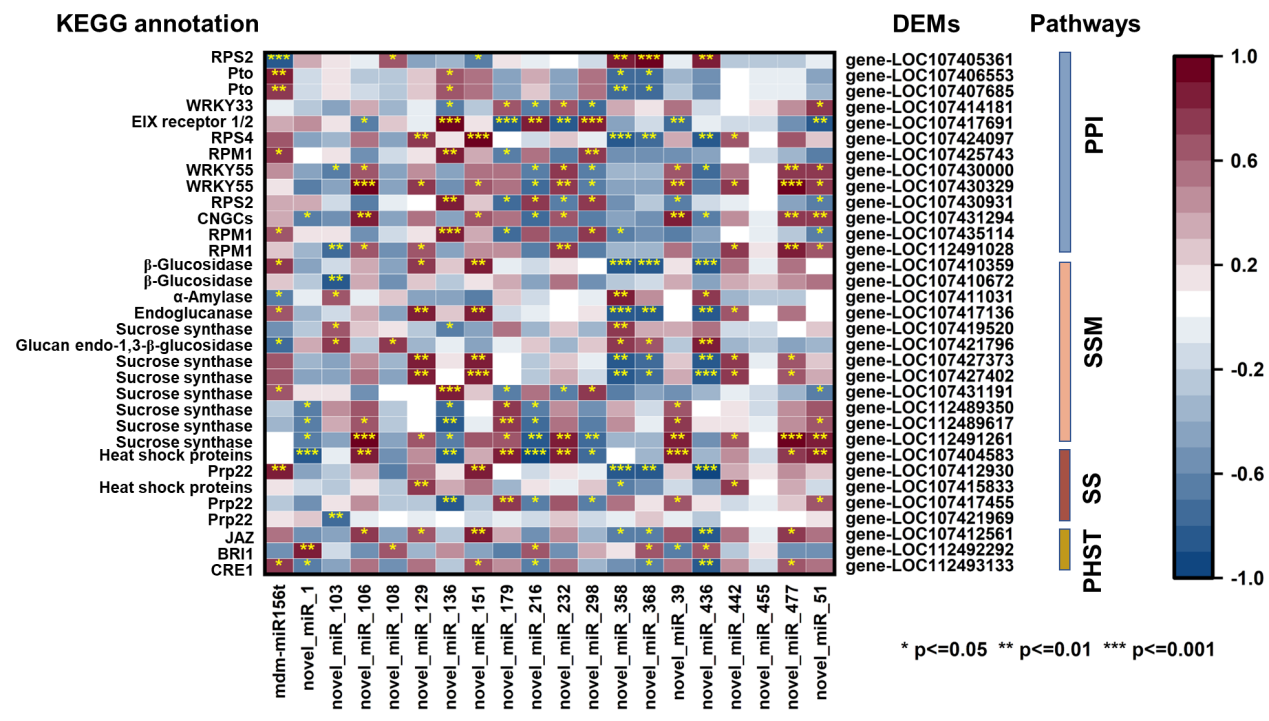


**Fig.S2** Heatmap analysis of the Person correlation between differentially expressed miRNAs (DEMIs) and their targeted gene expression levels of the top four KEGG enrichment pathways during high-temperature stress of *Ziziphus jujuba* leaves. PPI, SSM, SS, and PHST represent the plant-pathogen interaction, starch and sucrose metabolism, spliceosome, and plant hormone signal transduction pathways, respectively. The differentially expressed genes were identified with log_2_|FC| ≥ 2 and *P* value ≤ 0.05.





**Fig.S3** qRT-PCR validation. (A) RNA-seq results of six DEGs. (B) qRT-PCR results of six DEGs. The horizontal axis represents 0, 1, 3, 5, and 7 d of high-temperature stress (from left to right) of “Fucuimi” and “Junzao”, and the vertical axis represents the expression level. The white bar represents the expression level in 'Fucuimi', and the black bar represents the expression level in 'Junzao'. The error line indicates the SD (n = 3).
